# The manufacturing microbe

**DOI:** 10.1111/1751-7915.12183

**Published:** 2014-12-27

**Authors:** Willy Verstraete

**Affiliations:** LabMET, Ghent UniversityCoupure L653, Ghent, B-9000, Belgium

In all industrialized countries, the manufacturing industry appears to be in difficulty: the costs of maintaining skilled workers to do complicated technical tasks are becoming a major hurdle. The fact that a majority of the young professionals will spend their whole lives just typing in front of a screen is worrying plenty of sociologists, particularly because of the simple fact that our brains apparently are sparked to creativity by being involved in complex handling – read manufacturing – exercises (Sennett, 2014).

At this moment, the overall environmental biotech is undergoing a major shift, driven by the need to help to abate climate changes. Until now, it has been dealing with tasks of making unwanted compounds disappear: to degrade contaminants, to remove waste and to eliminate unwanted microbial propagules. In industrialized countries, this task is to a great extent achieved, and we now face a crucial challenge for our future: how to organize the circular economy. In other words, how to contribute to the recovery of resources from various ‘downstream’ products in a variety of anthropogenic-driven processes, simply formulated how to recover from various used materials and streams (Verstraete and Cornel, 2014). Clearly, as these ‘residuals’ are most often ‘mixed and full of disorder i.e. entropy’ and must be dealt with in a way that entails little costs, the need to use the intelligence of cheap labourers, i.e. the microbes, has become very prominent in this context of resource recovery.

There are already a number of niches where resource recovery manufacturing by means of microbial biotech has established a major foothold. Anaerobic digestion has some 30 000 industrial installations worldwide, totalizing a permanent power production equal to 10 000 MW. The total of the biogas plants represents the capacity of a nuclear power plant. The industrial recovery of sulfur is a well-established biotechnology nicely integrated in the petroleum sector (Janssen *et al*., 2001). The recovery of P by enhanced biological phosphorous removal is already implemented in many sewage treatment plants (Cornel and Schaum, 2009). There are several industrial technologies for the production of metals starting their recovery by bioconversion (Boonstra and Buisman, 2003).

However, there are some domains where microbial biotech is implemented in a way that is very questionable at the least. This year, we feature the fact that activated sludge exists as a technology for 100 years (Ardern and Lockett, 1914). Today, we must question this overall approach: we destroy organic matter at the expense of conventional energy and recover at the very best only a fraction of the energy present in the organics in the form of biogas. Moreover, in the conventional activated sludge process, basic nutrients such as nitrogen and phosphorous, as well as a primary source of life, i.e. water, are most often simply wasted. This technology has to be abandoned and must be replaced by an approach that is focused on the maximum recovery of organics and anorganics and particularly of the water, for instance by trapping the organics upfront and by making anaerobic digestion the major bioconversion process (Verstraete *et al*., 2009).

The second process which we should strongly reconsider is the application of nitrification and denitrification. By doing so, we use energy (both fossil and organic) to destroy a valuable resource: indeed 1 kg of mineral nitrogen requires some 2 kg of fossil fuel equivalent to produce. The fact that in the current ‘used’ water technology the general aspect of nitrogen ‘destruction’ at a cost of the order of 3–5 euro per kg of N is occurring without much questioning indicates that in the world of microbial biotech, a major wake-up call is necessary. Even the short circuit created in the nitrogen cycle by combining the ammonium to nitrite oxidation with the anammox conversion (the oxygen limited autotrophic nitrification denitrification process; Vlaeminck *et al*., 2012) is still a destructive process and although it comes at a cost of 1–2 euro per kg of N treated, its use should also be only advocated for cases where recovery is too minimal to be worth the effort. A bioprocess in the domain of N treatment that is in line of a sustainable bio-economy is, for instance, the Cando process. In this approach, nitrous oxide is recovered and used as fuel to power the reforming of ammonia to hydrogen fuel (Babson *et al*., 2013; Scherson *et al*., 2013), and it is of interest to see how these lines of development will work out.

Yet there is another domain of microbial biotech where the ‘used nitrogen’ can be recovered. Indeed, in plenty of downstreams (formerly referred to as wastewaters), the nitrogen is still in an organic form and can, by means of high loaded biosorptive sludge, be upgraded to microbial cells. The latter can subsequently be harvested and further processed as proteinaceous microbial biomass. In recent years, the prices of most food and feed commodities have been quite stable, whereas the price of protein (particularly of animal origin such as fish, milk and egg) has been rising. The prospects are that this trend, in view of the increase of proteinaceous food consumption worldwide, will not change since the surfaces to grow plant biomass are not increasing worldwide. Clearly, single-cell protein production, either for feed (for example in the aquaculture loop) or directly for food production (e.g. Quorn type of ingredients), deserves to be revisited from a variety of angles. A most intriguing perspective in this context is the use of renewable energy to hydrolyse water to hydrogen and oxygen, and to subsequently use the latter molecules to recapture reused ammonia, phosphorous and minerals (by means of hydrogen oxidizing bacteria) to make new food in a way that is ethically and aesthetically convenient.

Obviously, there are many more ways in which we can use the services of the manufacturing microbe to deal with the circular economy. The clever use of bio-electrochemistry offers perspectives to produce and harvest a variety of commodities such as hydrogen gas, sulfur, soda, peroxide, fatty acids and their derivatives [chemicals from residual streams (Rabaey and Rozendal, 2010)]. Doubtless, there is plenty of room for innovation in this domain.

Overall, the major challenge in using the capabilities of microbial biotech to participate in effective resource recovery is that we need young professionals adequately trained in the wheeling and dealing of these special biocatalysts. In this respect, we must advocate that training of scientists and engineers also keeps up the status of ‘hands on’. The latter, in view of the current overemphasis on the value of publications, has indeed suffered respectability and overall appraisal in the last decades. It must be clear that the manufacturing microbe needs the skills of the well-trained microbial biotech specialist.

To deal with sustainability and resource recovery, there will be a growing need for inventive processes, and it is clear that these processes will need skilled teams of people and microbes because they must start from materials which are complex and variable. By combining proper human resource management and microbial resource management, there is hope for effective overall resource recovery and lots of job opportunities for our young microbial biotech professionals.

## Conflict of interest

None declared.
